# Small but Mighty: Nanobodies in the Fight Against Infectious Diseases

**DOI:** 10.3390/biom15050610

**Published:** 2025-04-23

**Authors:** Wenning Jiang, Chundong Huang, Serge Muyldermans, Lingyun Jia

**Affiliations:** 1 Department of Public Security Administration, Liaoning Police College, Dalian 116036, China; 2 Dalian Kangyuan Medical Technology Co., Ltd., Dalian 116014, China; 3 Laboratory of Cellular and Molecular Immunology, Vrije Universiteit Brussel, Pleinlaan 2, 1050 Brussels, Belgium; 4 The School of Bioengineering, Dalian University of Technology, Dalian 116036, China

**Keywords:** nanobody, infectious diseases, virus, bacteria, treatment

## Abstract

Infectious diseases, caused by pathogenic microorganisms and capable of spreading, pose a significant threat to global public health. Developing efficient and cost-effective techniques for treating infectious diseases is crucial in curbing their progression and reducing patients’ morbidity and mortality. Nanobodies (Nbs), a novel class of affinity reagents derived from unique heavy chain-only antibodies in camelids, represent the smallest intact and fully functional antigen-binding fragments. Compared with conventional antibodies and their antigen binding fragments, Nbs offer numerous advantages, including high affinity, exceptional target specificity, cost-effective production, easy accessibility, and robust stability, demonstrating immense potential in infectious disease treatment. This review introduces Nbs and focuses on discussing their mechanisms and intervention strategies in the treatment of viral and bacterial infections.

## 1. Introduction

Infections in humans are among the most threatening public health challenges [1]. Several factors, including climate change, unprecedented population growth, and political instability, contribute to the increasing vulnerability to infectious diseases, globally [2]. Both unprecedented and re-occurring infections that once were controlled are appearing or re-emerging [3,4]. In the past two decades, in addition to the severe fever with thrombocytopenia syndrome virus [5] and Middle East respiratory syndrome coronavirus (MERS-CoV) [6], there have been outbreaks of Zika virus [7], yellow fever [8], and Ebola [9]. The novel coronavirus COVID-19, which exploded in 2020, has caused over 18.2 million deaths, worldwide [10]. While antibiotic therapy is one of the most common approaches for the treatment of bacterial infection, its excessive use has exacerbated the resistance of pathogens and compromised treatment effectiveness [11]. Therefore, it is imperative to develop more efficacious strategies for the control and mitigation of infectious diseases.

Antibodies (Abs) are considered as potential agents to prevent and treat human infections [12]. Although a vast number of monoclonal antibodies (mAbs) have been introduced on the market as diagnostics and therapeutics, only 8% of mAbs are used to combat infectious diseases, which is significantly lower than the percentage used for cancer treatment (45%) [13]. Many obstacles have impeded the utilization of mAbs in infection treatments, such as lengthy screening processes, high production costs, low stability, and large size [14].

Nanobodies (Nbs) are single-domain variable fragments of heavy chains of heavy chain-only antibodies circulating in camelids [15]. They have emerged as an alternative to conventional mAbs or smaller antigen-binding fragments derived from mAbs [16]. The molecular weight of Nbs is only 1/10 of that of conventional mAbs, making them the smallest molecular affinity reagent with complete antigen recognition functionality [17]. Additionally, Nbs are successfully expressed in high yields in prokaryotic systems and possess unique biochemical properties, such as high specificity, high stability, and ease of modification, demonstrating an immense potential to treat infectious diseases. This review begins by introducing the viral and bacterial pathogenic mechanisms of infectious diseases and then elucidates the possible intervention strategies of Nbs to treat infections. Subsequently, outlooks and future perspectives are discussed.

## 2. Characteristics and Preparation of Nanobodies

**Figure 1:**
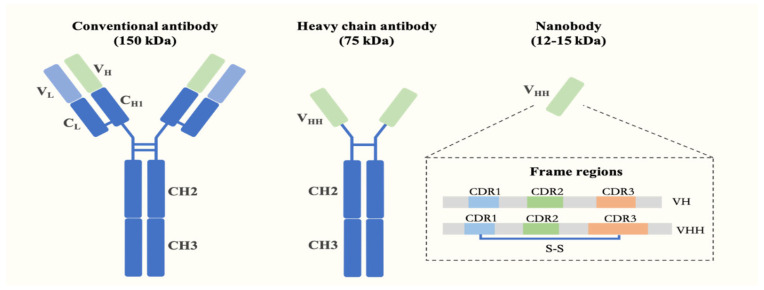
Structure of conventional, heavy-chain-only antibodies and nanobodies.

Conventional antibodies (relative molecular mass of ~150 kDa) are Y-shaped proteins, composed of two identical heavy chains and two identical light chains. In 1993, Hamers-Casterman et al. first reported the presence of naturally occurring heavy chain-only antibodies (HCAbs) in camel serum [18]. In 1995, Greenberg et al. also discovered similar HCAbs (known as IgNAR) in cartilaginous fish [19]. Nanobodies are recombinant antigen binding fragments derived from the variable domain of HCAbs (VHH). They have a molecular weight ranging from 12 to 14 kDa and an approximate diameter of 2.5 nm by 4 nm in length [17]. The antigen-binding region of Nbs consist of three complementarity determining regions (CDR1-CDR3, Figure 1). The CDR3 loop of Nbs plays a crucial role in antigen recognition. Hence, the average length of the CDR3 loop region of Nbs is on average significantly longer than the corresponding loop in human or mouse Abs [20,21]. This on average longer loop length allows the formation of new finger-like structures providing a larger interaction surface area to recognize the cognate antigen. Due to their reduced volume and size and the elongated CDR3 loop, Nbs exhibit a significant advantage in binding cryptic antigen epitopes, such as cavities or concave surfaces on pathogenic antigens, that are challenging targets for conventional mAbs [22].

In addition, the frequent presence of a disulfide bond within the CDRs of Nbs originating from camel HCAbs (Figure 1) can significantly enhance the structural rigidity of Nbs, thus conferring exceptional heat resistance and chemical tolerance [23]. Owing to their remarkable stability, Nbs exhibit sustained target-neutralizing activity even after aerosolization and lyophilization, making them a promising inhalable nebulizer for the prevention and treatment of severe air-borne infections [24,25]. Moreover, Nbs possess a higher abundance of hydrophilic amino acids, thereby demonstrating enhanced solubility in aqueous solutions. Nbs can be manufactured on a large scale utilizing easily reproducible expression systems such as *Escherichia coli* or yeast, at a cost well-below $100 per gram.

Overall, the production of nanobodies involves three steps: (i) construction of a Nb-gene library, (ii) enrichment, retrieval, selection, and identification of the target-binding Nbs, and (iii) their recombinant expression. Nanobody gene libraries can be classified into immune libraries, and non-immune (either naïve or synthetic) libraries [26]. Among them, immune libraries, constructed from the lymphocytes of an immunized camelid (or HCAb transgenic mouse), are the most commonly used and advantageous for obtaining Nbs of highest specificity and affinity for their cognate target [27]. After amplifying and inserting the Nb genes into dedicated cloning vehicles, specific antigen-binding Nbs can be further enriched using bio-panning techniques such as phage display [28]. Currently, researchers have reported Nbs against over 1000 antigens using immune libraries and phage display selection technologies [29]. Finally, dedicated microorganism expression systems and purification systems are employed to obtain Nbs that can be used in downstream applications. Protein engineering techniques have also been developed to mutagenize the CDR regions of Nbs to further improve Nb affinity or specificity [30]. The detailed Nb identification and production process has been summarized in several recent reviews [16,28,31].

## 3. Viral Infection Pathways and Possible Intervention Strategies with Nbs

**Figure 2:**
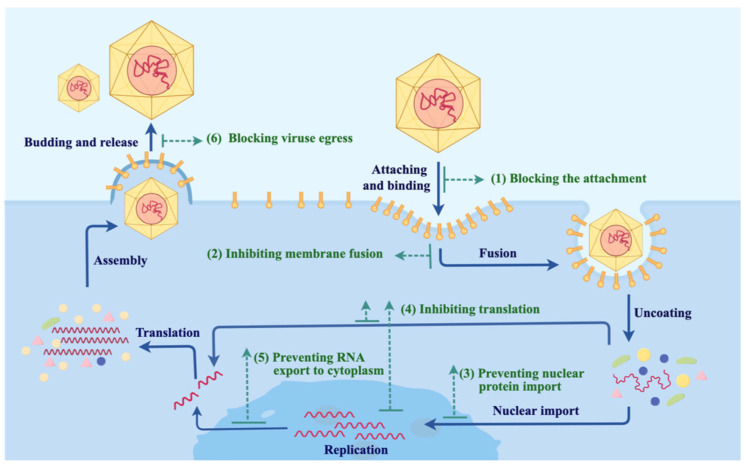
Mechanisms of viral infection and the versatile applications of nanobodies. (1) Blocking virus–host recognition by targeting either viral protein or a cellular protein. (2) Inhibiting membrane fusion and thus inhibiting viral particle penetration into host cells. (3) Preventing the nuclear import of the viral genome and thus inhibiting viral replication (4) Inhibiting viral protein translation. (5) Preventing viral RNA export to the cytoplasm. (6) Blocking the egress of mature viruses from infected cells.

The virus life cycle in humans mainly includes stages such as attachment and binding to the host cell surface, penetration into host cells, genome uncoating, genome synthesis and replication, virus assembly, and escape from host cells (Figure 2) [32]. Nbs are capable of exerting antiviral effects at various stages of the viral life cycle. For example, they can target the viral surface proteins involved in host cell recognition, thereby blocking the binding and fusion between the virus and host cell membranes and inhibiting the invasion of host cells. The presence of intracellular Nbs might also inhibit genome nuclear import, replication, and transcription processes within host cells, thus decreasing the viral load. Furthermore, Nbs can prevent the egress of mature viruses, inhibiting the dissemination to new cellular targets. In this section, we focus on the current progress and typical roles of Nbs in viral infection treatment.

### 3.1. Blocking Virus–Host Recognition and Membrane Fusion

The recognition of host cell receptors represents a critical initial step in the viral infectious life cycle and plays a key regulatory role in viral pathogenesis. Owing to their high affinity and strong specificity, nanobodies offer substantial advantages in blocking viral–host cell recognition and inhibiting the membrane fusion process.

Respiratory syncytial virus (RSV) is a prominent pathogen responsible for acute lower respiratory infections (ALRIs) in young children [33]. The F glycoprotein on the surface of RSV virions facilitates fusion between the virus and host cells, thus serving as a key factor in the treatment of RSV infection [34]. In 2016, Ablynx retrieved a monovalent Nb017 nanobody targeting the antigenic site II of the RSV F glycoprotein from a llama immune library. Subsequently, they employed glycine–serine (GS) linkers to construct a trivalent nanobody known as ALX-0171. The trivalent ALX-0171 antibody exhibited a significantly enhanced apparent affinity, surpassing that of the monoclonal antibody Nb017 by over 160-fold. Moreover, it demonstrated an impressive viral replication blocking efficacy of 87%, far exceeding the FDA-approved palivizumab antibody (18%). By nebulizing a low dose of ALX-0171 (1 mg/kg) directly into the rat nasal canals and lungs, near-complete suppression of RSV virus replication in the murine lungs could be achieved. In a Phase 2b clinical trial conducted in 2021 [35], ALX-0171 treatment led to an effectively reduced viral load, thereby demonstrating substantial anti-RSV activity and demonstrating its promise as a viable therapeutic approach for managing RSV disease. However, ALX-0171 did not yield significant improvements during RSV infection among hospitalized children. A comprehensive investigation into the structure and functionality of the RSV F glycoprotein discovered that targeting the prefusion conformation of the F glycoprotein (PreF) was a more efficacious antiviral strategy owing to its fusion-competent nature and being the primary target of neutralizing activity found in human serum [22,36,37]. Rossey et al. [36] reported two llama-derived nanobodies (F-VHH-4 and F-VHH-L66) that recognized a conserved cavity formed by two F protomers with a high affinity of 18 pM, representing promising antiviral agents against RSV. Subsequently, they identified a PreF-specific nanobody named as F-VHH-Cl184, which predominantly bound to a unique epitope located in the lower region of PreF. Structural analysis revealed that two hydrophobic residues of F-VHH-Cl184 CDR3, Val100 and Trp100e are neatly packed in a small cavity between antigenic sites I and IV, and Glu100d forms a salt bridge with Lys465 at β22.Consequently, it is hypothesized that F-VHH-Cl184 neutralized RSV by stabilizing β22 in its prefusion conformation and thereby preventing its transition into post-fusion F [37].

Coronaviruses (CoVs) are enveloped viruses with a linear, single-stranded positive-sense RNA genome. Notable members of CoVs include the Middle East Respiratory Syndrome Coronavirus (MERS-CoV), Severe Acute Respiratory Syndrome Coronavirus (SARS-CoV-1), and the recently identified SARS-CoV-2 [38]. The receptor-binding domain (RBD) within the spike protein S1 subunit of coronaviruses facilitates viral entry into host cells by binding to surface receptors, such as angiotensin-converting enzyme 2 (ACE2) [39]. In 2020, Wrapp et al. [40] identified two potent neutralizing Nbs derived from llama HCAbs, showing cross-reactivity with RBDs of both the SARS-CoV-1 and SARS-CoV-2 spike proteins. This discovery has sparked a surge of research interest in harnessing Nbs for the prevention and treatment of coronaviruses. Dozens of Nbs have been reported to selectively target the S protein RBD domain [24,40,41,42,43,44,45,46,47,48,49,50,51,52,53,54,55,56], thereby impeding the interaction between the S protein and ACE2 receptor on host cellular surfaces. Researchers have successfully developed efficient libraries for the generation of SARS-CoV-2-specific humanized Nbs, and have identified several humanized Nbs that target multiple cryptic epitopes located within the RBD region [48,50]. Custódio et al. [49] demonstrated that the small size and high affinity of Nbs facilitated their binding to distinct regions of the RBD from diverse orientations. In contrast to mAbs that exclusively bind to the RBD in the “up” conformation, the isolated Sb23 Nb demonstrated binding affinity for both “up” and “down” conformations of the RBD, thereby highlighting their potential efficacy in blocking ACE2 binding. By screening a yeast surface-displayed library of synthetic nanobody sequences, Schoof et al. [56] successfully developed a dual-functional nanobody, Nb6, that specifically bound to the inactive “down” conformation of RBD. Cryo-electron microscopy (cryo-EM) analysis disclosed that Nb6 binds to the closed spike protein by straddling the interface between two neighboring RBDs. The CDR1 and CDR2 of Nb6 contribute the majority of the contacting surfaces, and the CDR3 contacts the adjacent RBD in a counterclockwise direction when viewed from the top. Consequently, a single Nb6 molecule stabilizes two adjacent RBDs in the down state. This stabilization may also preorganize the binding site, facilitating the binding of subsequent Nb6 molecules and further stabilizing the closed spike conformation. Due to their reduced size and absence of Fc tails, Nbs exhibit a lower immunogenicity compared to mAbs, which is unfavorable for triggering the immune system to kill pathogens. Thus, by conjugating the selected Nbs with the human IgG1 Fc domain, the modified Nbs yielded superior virus neutralizing capacity compared to the primitive ones [57]. In addition to inhibiting the ACE2-RBD interaction, nanobodies can also disrupt the fusion of viral and host membranes. The unmodified nanobody, K-874A, as reported by Haga et al. [58], exhibited a strong binding affinity towards the receptor binding domain and N-terminal domain of the S protein. This interaction effectively impeded viral membrane fusion and hampered viral replication within host cells. The S2 subunit of the S protein is responsible for membrane fusion and exhibits greater conservation among SARS-CoV-2 variants, highlighting its significance in viral entry and potential as a therapeutic target [59]. Recently, Feng et al. [60] identified a nanobody derived from sharks, namely 79C11, which specifically binds to the highly conserved HR1 region in the S2 domain. As a result, it demonstrates broadly neutralizing activities against a wide range of SARS-CoV-2 variants. To achieve the robust production of nanobodies for SARS-CoV-2, Zhao et al. [61] established a visually trackable and highly efficient secretory expression system for RBD-specific Nbs, which was realized by fusing the super-folder green fluorescent protein (sfGFP) to either the N-terminus or the C-terminus of the Nb.

Influenza virus is a predominant pathogen responsible for respiratory infections in humans, imposing a significant disease burden and associated mortality. Influenza A virus (IAV) and influenza B virus (IBV) are the primary causative agents of seasonal flu outbreaks. The hemagglutinin protein (HA), known as the major surface glycoprotein of influenza virus, is responsible for receptor binding and viral infection; it forms a homotrimer with each monomer composed of two subunits referred to as HA1 and HA2 [62]. The globular head domain (HA1) is a critical antigenic epitope that mediates the binding of sialic acid receptors on host cells. However, due to the high variability of the HA head, the Nbs targeting the HA head generally exhibit limited cross-reactivity against antigenically drifted viral strains [63,64]. For example, Ramage et al. [65,66] reported Nbs named Vic2a-6 and YamNGS#1 that specifically targeted the head domain of IBV. However, their neutralizing activity was limited to the B-Victoria and B-Yamagata lineages, respectively, indicating a restricted cross-reactivity. Shcheblyakov et al. [67] identified three H3-specific VHHs that bind to the HA head of different H3N2 strains. They further expanded the binding spectrum and activity of these VHHs by fusing them to the Fc region. To achieve broad protection against influenza viruses, the conserved epitopes of the HA head have drawn extensive attention in vaccine development [68]. Barbieri et al. [69] identified the E13 Nb, which recognizes highly conserved conformational epitopes on the H1 HA1, which significantly inhibited the replication of a panel of H1N1 strains that have undergone over 80 years of antigenic drift. Recently, Chen et al. [70] isolated a H7-HA-specific Nb named E10, which targets the conserved lateral region of the HA head, thereby achieving broad-spectrum binding and cross-group neutralization, and providing in vivo protection against various IAV subtypes. The HA stem region, mainly composed of HA2, exhibits higher conservation across different influenza subtypes, making it a pivotal target for the development of universal influenza vaccines. Voronina et al. [71] reported two high-affinity neutralizing Nbs specifically targeting the HA stem region of the IAV virus, enabling the complete neutralization of the H1N1 and H5N2 viruses in vivo. Hufton et al. [72] reported a HA stem region-targeting R1a-B6 Nb from an immune alpaca library, which exhibited broad-spectrum neutralizing activity against the H1N1, H5N1, and H9N2 strains.

In addition to viral surface glycoproteins, host cell surface receptors are also important targets for inhibiting virus binding and infection. Human immunodeficiency virus (HIV) poses a significant threat to human health by impairing the normal functioning of the host’s immune system through binding to CD4 receptors located on the surface of T lymphocytes. The chemokine receptors CXCR4 and CCR5 are predominantly expressed in monocytes, neutrophils, and activated T lymphocytes. They function as the primary co-receptors facilitating HIV virus entry into immune cells and have received significant attention in the field of HIV treatment. Jähnichen et al. [73] demonstrated that Nbs targeting the cell receptor CXCR4 could effectively prevent HIV infection of host cells. However, CXCR4 also functions as a receptor for the chemokine CXCL12, and the signaling mediated by the CXCR4/CXCL12 axis plays a pivotal role in cellular migration and normal immune function. Consequently, precise targeting of the HIV binding region on CXCR4 while preserving its interaction with CXCL12 is imperative within the realm of HIV treatment. Van Hout et al. [74,75] isolated a series of nanobodies that could specifically recognize CXCR4 and evaluated their modulating downstream signaling pathways in host cells. Epitope mapping analysis revealed the VUN402 Nb selectively targeted the HIV-binding site, thereby impeding viral entry into cells while preserving normal CXCR4 receptor binding to CXCL12 and the subsequent signal transduction. Furthermore, the CXCR4 Nbs could be utilized as a drug carrier for the targeted delivery of various therapeutic agents. For instance, Cunha-Santos et al. [76] successfully combined the CXCR4 receptor-targeting nanobody with small interfering RNA (siRNA) to achieve specific siRNA delivery into T lymphocytes expressing CXCR4, resulting in the effective suppression of HIV virus transcription and a reduction in replication levels.

Currently, owing to the high sensitivity and multi-epitope recognition advantages of membrane proteins, Nbs have exhibited potent virus neutralization effects in in vitro and in vivo experiments against a range of emerging and re-emerging viruses, including the poliovirus virus [77,78,79], hepatitis B virus [80], hepatitis C virus [81], human papillomavirus virus [82], rotavirus virus [83], Ebola virus [84], bunyaviridae virus [84], chikungunya virus [85,86,87], nipah virus [88], and thrombocytopenia syndrome virus [89] (Table 1).

### 3.2. Inhibiting Intracellular Viral Replication and Transcription

The nuclear protein (NP) is a structural protein inside the influenza virus, which can facilitate the entry of the virus genome into the host cell nucleus by recognizing nuclear localization signals. Thereby, the NP plays a crucial role in the replication cycle of the virus. Ashour et al. [105,106] isolated Nbs named αNP-VHHs that could specifically recognize the NP in living cells. αNP-VHHs exhibited the ability to bind with NPs within the cytoplasm, thus impeding virus genome entry into the host cell nucleus and disrupting virus replication at an early stage of the life cycle.

The HIV-1-encoded regulator of virion expression (Rev) is indispensable for the expression of late viral mRNAs. Rev facilitates the nuclear–cytoplasmic transport of viral mRNA by interacting with the Rev response element (RRE) of viral pre-mRNA species. Vercruysse et al. [112] reported the selection of Nb190 from immune llama, which specifically bound to the N-terminal helical multimerization domain (K20 and Y23 epitopes) of the Rev protein, thus inhibiting the oligomeric assembly of REV and reducing its affinity for RRE. Consequently, cells stably expressing Nb190 were protected against virus-induced cytopathic effects by inhibiting HIV-1 replication and specifically suppressing the Rev-dependent expression of partially spliced and unspliced HIV-1 RNA [115].

The multifunctional accessory protein Nef is important for the complete virulence of HIV-1, exhibiting diverse roles in HIV pathogenesis including CD4 downregulation, major histocompatibility complex I downregulation, activation of p21-activated protein kinase (Pak2), and augmenting of virion infectivity [116]. Bouchet et al. [113,117] reported Nef-targeted Nbs that effectively inhibit the critical biological activities of Nef both in vitro and in vivo.

Nonstructural protein 3 (NS3) is an essential enzyme of the hepatitis C virus (HCV) replication complex. Phalaphol and Jittavisutthikul et al. [107,108] reported a set of Nbs that enables binding to the residues of the protease catalytic triad. Modified with a 16 amino acid cell penetrating peptide (CPP), the NS3-targeted cell penetrable Nbs might not only inhibit the helicase activity involved in RNA unwinding and replication, but also disrupt the NS3–core interaction, thereby affecting virus morphogenesis and leading to a reduction in virus egress.

### 3.3. Inhibiting the Egress of Newly Made Viruses

Neuraminidase (NA), another prominent surface glycoprotein of the influenza virus, is capable of cleaving sialic acids from cellular receptors [118]. The cleavage of sialic acids by NA prevents the aggregation of virions and inhibits their binding to dying host cells through HA, facilitating efficient the egress of viral particles and dissemination to new cellular targets. Cardoso et al. [103] successfully isolated H5N1 NA-specific Nbs, which effectively inhibited the NA activity and the replication of clade 1 and clade 2 H5N1 viruses.

The ion channel protein M2 on the surface of the influenza virus plays a crucial role in the process of viral uncoating, assembly, and budding [119]. Wei et al. [104] demonstrated the potent inhibitory effects of M2 protein-targeted Nbs on the infection and replication capacity of the H3N2 and H1N1 viruses by interference with their ion channel function. The NA and M2 proteins exhibit highly conserved sequences across various subtypes of influenza A virus, making them an appealing target for broad-spectrum protection. However, due to the relatively lower immunogenicity of these proteins, further investigation is warranted to identify high-affinity Nbs that specifically target NA and M2.

## 4. Mechanisms and Intervention Strategies of Nbs in Bacterial Infectious Treatment

Bacterial infections cause serious illnesses that can lead to gastrointestinal disturbances, inflammation, systemic infection, and potentially fatal outcomes. It is projected that the annual mortality rate of bacterial infections might reach 10 million by 2050 [120]. Antibiotics have traditionally been widely used and effective in treating bacterial diseases. However, since multiple bacteria have developed drug resistance levels exceeding 50%, there is an urgent need for a more powerful treatment method [121]. Nanobody-based antimicrobial drugs aim to address bacterial resistance while preserving healthy human microbiota, introducing a novel antibacterial treatment strategy with high sensitivity and specificity.

**Figure 3:**
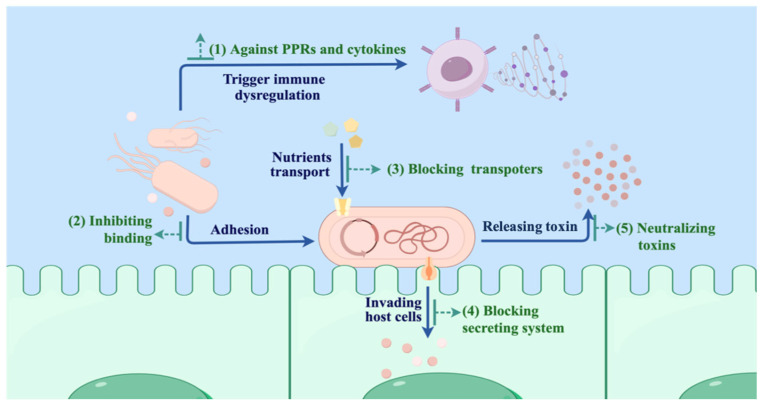
Mechanism of bacterial infection and the versatile applications of Nbs. (1) Combating host pattern recognition receptors (PRRs) and targeting excessive cytokines. (2) Inhibiting bacterial adhesion and colonization. (3) Blocking nutrient transporters, thereby inhibiting bacterial growth. (4) Inhibiting bacterial invasion by blocking the bacterial secreting system. (5) Neutralizing the released toxins.

Nanobodies exhibit diverse applications in the diagnosis and treatment of both Gram-positive and Gram-negative bacteria [122]. This review focuses on the therapeutic applications of nanobodies in highly lethal and strongly drug-resistant bacterial infectious diseases. When a bacterial pathogen encounters a human host, the lipopolysaccharide (LPS), peptidoglycan, lipoteichoic acid (TLA) and other pathogen-associated molecular patterns (PAMPs) of bacteria may trigger dysregulation of the immune system [123,124], resulting in persistent inflammatory responses and excessive cytokine release [125], thereby provoking severe conditions such as sepsis [126]. In addition, bacteria with adhesins can bind to and colonize host cells, and further invade host cells and release toxins. (Figure 3). Currently, the intervention strategies of nanobodies focus on inhibiting bacterial adhesion, colonization, and growth, blocking bacterial invasion, neutralizing released toxins, as well as combating host pattern recognition receptors (PRRs) and targeting excessive cytokines.

### 4.1. Inhibiting Bacterial Adhesion, Colonization, and Growth

Enterotoxigenic *Escherichia coli* (ETEC)-induced diarrhea remains a prominent cause of mortality among children under 5 years of age in developing countries. ETEC strains cause diarrhea by adhering to enterocytes in the small intestine using one or more adhesins known as colonization factor (CF) antigens. ETEC strains exhibit antigenic diversity with more than 25 types of CF and coli surface (CS) antigens being identified. Therefore, developing broad-spectrum ETEC vaccines and therapeutic drugs poses significant challenges. Amcheslavsky et al. [127] immunized llamas with human specific CFs, and subsequently identified four nanobodies through yeast display technology. Notably, 2R215 Nbs recognized a highly conserved epitope within the putative receptor binding region of ETEC adhesins, thus demonstrating cross-protective potency against eleven pathogenic ETEC strains in vitro and resulting in a significant reduction in the total bacterial count in vivo. This study holds great significance for the prevention of human ETEC infections.

*Acinetobacter baumannii* is a major genus of nosocomial infections that can tolerate high acidity and alkalinity, high temperature, common medical disinfectants, and ultraviolet radiation. The mortality rate of *A. baumannii* is as high as 45%, making it one of the most dangerous strains to human health. The biofilm-associated protein (Bap) on the surface of *A. baumannii* is composed of tandem repeat sequences, playing a vital role in biofilm formation and intercellular adhesion, which are associated with bacterial virulence and drug resistance. Payandeh et al. [128] reported Nbs that specifically targeted the conserved region of Bap. These Nbs were capable of inducing conformational changes in the Bap protein, which leads to the inhibition of biofilm formation and bacterial adhesion. Knauf et al. [129] identified several *A. baumannii*-targeting Nbs through generation and panning of a synthetic nanobody library.

*Pseudomonas aeruginosa* is another leading cause of nosocomial infections in immunocompromised individuals, including those with burn injuries. Adams et al. [130] immunized llamas with outer membrane proteins of *P. aeruginosa* and successfully selected a specific nanobody, 9D, which selectively targeted the type-a flagellin protein of *P. aeruginosa*. This 9D nanobody exhibited inhibitory effects on the motility and biofilm formation of type-a *P. aeruginosa* in vitro. However, it did not possess broad-spectrum inhibitory activity against *P. aeruginosa* due to its inability to recognize the type-b flagellin protein.

Bacterial ATP-binding cassette (ABC) importers catalyze the uptake of essential nutrients including transition metals and metal-containing co-factors, which play important roles in bacterial colonization and growth. Mireku et al. [131] reported a Nb9 that specifically targeted the BtuCD-F transporter of *E. coli*. The crystal structure of Nb9-BtuF revealed that Nb9 bound to BtuF by inserting CDR3 into the deep Cbl-binding cleft. Inhibiting by 70% the transport activity of vitamin B12 to BtuCD-F, Nb9 effectively hindered the uptake of essential nutrients, thus providing a novel antibiotic strategy.

### 4.2. Blocking Bacterial Invasion

The secretion of bacterial proteins across host cell membranes constitutes a crucial aspect of bacterial invasion in mammalian hosts. Pathogens utilize dedicated protein secretion systems to secrete virulence factors from the cytosol of bacteria into host cells or the host environment. Given the specific expression patterns of certain secretion systems in bacterial pathogens, efforts are underway to develop antimicrobials targeting these systems as a means to enhance our existing arsenal of antibiotics. The dedicated secretion systems in Gram-negative bacteria are numbered type I through type VI, with each system transporting a specific subset of proteins [132].

Type III secretion systems (T3SSs) are found in a large number of Gram-negative bacterial pathogens and symbionts [133]. The translocated intimin receptor (Tir) is one of the effector proteins translocated by *E. coli*’s T3SS into host cells. After folding, Tir integrates into the host membrane to provide a pedestal for adhesion via intimin binding. Kühne et al. [134] reported several Nbs that can recognize different C-terminal epitopes of *E. coli* intimin, providing novel targets for disrupting bacterium–host cell interaction and therefore disease resistance strategies.

Type IV secretion systems (T4SSs) are ancestrally related to bacterial DNA conjugation systems and can secrete a variety of substrates, including single proteins and protein–protein and DNA–protein complexes [135]. *Ehrlichia* translocated factor-1 (Etf-1), a T4SS effector, is a primary virulence factor for *Ehrlichia chaffeensis*. Zhang et al. [136] successfully identified NbD7, which specifically inhibited three activities of Etf-1. By conjugating to cyclized cell-permeable peptide 12 (CPP12), the CPP12-NbD7 effectively entered mammalian cells and abrogated the blockade of cellular apoptosis caused by *E. chaffeensis* and inhibited infection in cell culture and in a severe combined-immunodeficiency mouse model. These findings suggest the potential of Nbs as a therapeutic intervention for human monocytic ehrlichiosis and other intracellular infections.

The type VI secretion system (T6SS) is a multicomponent nanomachine of bacteria that is analogous to the contractile bacteriophage tail, which directly injects effector molecules into host cells and disrupts cellular functions. T6SS is composed of a cell envelope-associated membrane complex, which spans both membranes and assembles from TssJ, TssL, and TssM [137]. Nguyen et al. [138,139] reported a TssM-targeted Nb25, which effectively inhibited the toxin secretion function of T6SS by preventing TssM-TssJ complex formation.

### 4.3. Neutralizing Bacterial Toxins

Shiga toxin-producing *Escherichia coli* (STEC) is a zoonotic pathogen that poses significant threats to both human and animal health. STEC is considered one of the key foodborne microorganisms responsible for global food safety incidents, which are frequently associated with severe forms of infection including hemorrhagic colitis and hemolytic uremic syndrome [140]. Shiga toxins are broadly classified into two types: Shiga toxin1 (stx1) and Shiga toxin 2 (stx2). Lo et al. [141] reported a NbStx2e1 Nb that targets the B subunit of the Stx2e toxin. Structural analysis revealed that for each B subunit of Stx2e, one NbStx2e1 interacted in a head-to-head orientation and directly competed with the glycolipid receptor binding site on the surface of the B subunit. The neutralizing NbStx2e1 Nb demonstrated a potent ability to neutralize the toxin and shows promise for the prevention or treatment of edema disease. Tremblay et al. [142] reported a “VHH-based neutralizing agent” (VNA) that prevented death or kidney damage in mice following challenge with Stx1 or Stx2. A single VNA, consisting of a double-tagged VHH heterotrimer, one specific for Stx1, one specific for Stx2, and one cross-specific for both Stx1 and Stx2, was effective in preventing all symptoms of intoxication from Stx1 and Stx2. In comparison to monovalent or bivalent Nbs, the VNA demonstrated an approximately two-fold decrease in IC50 and exhibited enhanced viral neutralization effects. Furthermore, Robinson et al. [143] identified several Stx2-neutralizing Nbs with good affinity for Stx2d and created a Nb heterotetramer (VNA2-Stx) for broader Stx natural variant specificity. In addition, they utilized self-amplifying replicon RNA (repRNA) to encode VNA2-Stx and administered it to the mouse model via intramuscular injection.

*Clostridium difficile*-associated diarrhea (CDAD) is the most common cause of infectious diarrhea associated with exposure to healthcare settings [144]. Pathogenic *C. difficile* strains [145] cause diarrhea and colitis with a high recurrence rate by releasing two toxins, enterotoxin (TcdA) and cytotoxin (TcdB). TcdA and TcdB share homology and possess similar structural modules: an N-terminal glucosyltransferase domain (GTD), followed by a cysteine protease domain (CPD), an intermingled membrane translocation delivery domain and receptor-binding domain (DRBD), and a large C-terminal combined repetitive oligopeptides domain (CROPS). The CROPS domain was once acknowledged as a pivotal site for the interaction between TcdA, TcdB, and receptors on the intestinal epithelial cell surface. While several Nbs with high affinity have been selected from the full-length CROPS [146], their ability to neutralize TcdB toxin in vitro was limited. The primary reasons are as follows: ① The CROPS domain harbors multiple binding sites for cellular receptors [147], and the selected Nbs [148] did not adequately target all these binding sites on the CROPS; ② Apart from the CROPS domain, both the upstream DRBD region [149] and coiled-coil protein can also interact with other receptors on host cell surfaces, thereby inducing cytotoxicity. Therefore, in recent years, researchers have been devoted to applying nanobodies for recognizing and neutralizing other structural domains of TcdB and TcdB toxins [150]. Yang et al. [148,151,152] developed a series of Nbs that specifically targeted the GTD, CPD, and DRBD of the TcdA and TcdB toxins. Furthermore, these Nbs were ingeniously linked to form a tetravalent Nbs named “ABA”, enabling simultaneous targeting of both the TcdA and TcdB toxins. A single injection of ABA at a dosage of only 3.2 µg/kg provided complete protection against lethal systemic toxin challenge in mice, which constituted merely 1/50 of the dosage of conventional mAbs. In addition, researchers have also demonstrated the potential application of Nb-expressing adenovirus [153], engineered lactobacillus [154], and probiotics [155] for the treatment of CDAD in murine models. *C. difficile* transferase toxin (CDT), which is considered as the third toxin expressed by some hypervirulent strains, plays a pivotal role in bacterial colonization and attachment. The CDT complex consists of the enzymatic ADP-ribosyltransferase (CDTa) and the binding component (CDTb). Unger et al. [156] selected a panel of Nbs that not only inhibited the CDTa-mediated ADP-ribosylation of actin but also mitigated the cytotoxicity of CDTb by specifically binding to its catalytic domain and impeding CDTa translocation.

*Bacillus anthracis*, the causative agent of anthrax, poses a significant bioterrorist threat as its spores can persist in the environment for an extended period and reactivate in the host. The anthrax toxins consist of three synergistically acting proteins: protective antigen (PA), edema factor (EF), and lethal factor (LF) [157]. PA is capable of mediating the entry of LF and EF into cells by binding to cell membrane receptors, making it a key target for anthrax therapy. Shali et al. [158] selected Nbs that specifically targeted the receptor binding domain (PAD4) of the PA toxin, thereby effectively preventing the binding of PA to cell receptors and inhibiting toxin internalization. Moayeri et al. [159] reported a heterodimer of Nbs named “VNA”, which can simultaneously target the receptor binding domain of the PA toxin and inhibited its self-assembly function. This study demonstrated the promising therapeutic efficacy of Nbs in an anthrax spore challenge model. In addition, Vrentas et al. [160] selected a new set of Nbs against anthrax toxins that act by binding to the EF and/or LF components, and reported a bispecific Nb multimer that effectively targets LF and EF toxins, demonstrating complete protection against lethal anthrax spore infection in mice as a single dose.

*Helicobacter pylori*, a bacterium capable of producing urease, is responsible for infecting approximately half of the global human population, thereby significantly elevating the susceptibility to peptic ulcers and gastric cancer. Ardekani et al. [161] isolated Nbs that target the UreC subunit of urease, which could inhibit urease activity and function. Hoseinpoor et al. [162] constructed a highly diversified phage-displayed VHH library using error-prone PCR technology and selected a mutant HMR2 Nb that targets the UreC subunit with superior inhibitory activity at low concentrations (Table 2).

### 4.4. Blocking Pattern Recognition Receptors

Pattern recognition receptors (PRRs) are a class of host receptors that play pivotal roles in recognizing conserved pathogen-associated molecular patterns (PAMPs), thereby initiating downstream signaling pathways [167,168]. This ultimately leads to the activation of interferon regulatory factors (IRFs) and facilitates the expression of proinflammatory cytokines [169]. As a member of the Toll-like receptor (TLR) family, Toll-like receptor 4 (TLR4) plays a crucial role in innate immunity and orchestrates inflammation through its recognition of lipopolysaccharide (LPS) or bacterial endotoxin [170]. The development of TLR4-targeted drugs can effectively inhibit the excessive production of various inflammatory factors [171]. Liao et al. [172] obtained Nbs that targeted C-terminal and intermediate domain of TLR4. Supported by in vitro and in vivo experiments, these Nbs demonstrated significant efficacy in attenuating the release of inflammatory factors and enhancing animal survival rates, thereby offering novel insights and strategies for the clinical management of sepsis. C-type lectins are endocytic receptors mostly expressed by macrophages, DCs, and some endothelial cells [173]. They contribute to innate and adaptive antibacterial immune responses by recognizing surface polysaccharides of specific pathogens [174]. Clec4f is a member of the type II C-type lectin family, playing a crucial role in α-galactose ceramide presentation as well as *Listeria monocytogenes* infection in mice [175]. Zheng et al. [176] developed a series of Nbs that target multiple distinct recognition epitopes of Clec4F, demonstrating great potential in molecular imaging [177] and therapeutic applications.

In recent years, researchers have increasingly focused on the application of Nbs in immune disorders like cytokine storms. For instance, Sparkes et al. [178] developed Nbs that specifically target macrophage migration inhibitory factor (MIF), thereby mitigating the inflammatory response and reducing mortality associated with bacterial toxins in murine models.

## 5. Conclusions and Prospects

Nanobodies, as a cost-effective and efficient alternative to traditional mAbs, offer a potent tool for the prevention and treatment of infectious diseases. According to the extensive research report by Market Research Future in 2022, it is projected that the nanobody market will attain a value of $1.14 billion by 2030, exhibiting a compound annual growth rate of 24.2%. The global nanobody market is anticipated to witness rapid expansion in the forthcoming years. Presently, the application of nanobodies in combating infectious diseases primarily revolves around disrupting pathogen life cycles and inhibiting viral replication. Currently, only three types of nanobody products have successfully completed phase II clinical trials for infectious diseases such as rotavirus, respiratory syncytial virus, and *Campylobacter jejuni*. In comparison to the well-established and extensively marketed antibody drugs, the translational progress of nanobodies is still in an initial stage (Table 3).

Due to the diverse subtypes of pathogenic antigens, the development of nanobodies with broad-spectrum capabilities for neutralizing pathogens still faces numerous challenges. Isolating universal nanobodies capable of recognizing highly conserved epitopes from multiple pathogenic antigens holds immense potential for broad-spectrum antiviral therapy [163]. Nbs offer significant advantages in targeting hidden and inaccessible conserved epitopes due to their small size and extensive CDR3 region. With the continuous development of computer simulation technology and protein structural elucidation techniques, researchers are poised to discover and optimize high-performance nanobodies capable of recognizing conserved epitopes.

The reduced size of Nbs offers significant advantages, including enhanced tissue penetration, the ability to traverse the blood–brain barrier, and access to challenging epitopes. However, this characteristic also leads to a shortened circulating half-life due to high clearance rates. To address these challenges and enhance the viability of Nb-based infectious therapy, various strategies have been developed. A key approach involves the construction of Nbs into bivalent and trivalent formats. Furthermore, some researchers have investigated the conjugation of selected VHHs with a specific VHH-targeting serum albumin. Mejías et al. [164] developed a trivalent (2vb27)2-SA nanobody by conjugating two Nbs targeting Stx2B with one Nb targeting serum albumin, which exhibited an extended half-life of 15 days in the bloodstream and achieved a 100% survival rate in a mouse model injected with a lethal dose of Stx2.

Furthermore, leveraging the inherent stability and facile expression of nanobodies, the utilization of nanobody-based oral and inhalation drugs for infectious diseases, along with employing nanobody therapeutic strategies within probiotic systems and adeno-associated virus (AAV)–gene vectors, will pave novel avenues in the realm of infectious disease prevention and treatment. Laursen [102] employed a recombinant adeno-associated virus (AAV) vector to effectively express influenza HA domain-targeted Nbs in murine nasopharyngeal mucosal cells, resulting in an extended duration of viral prophylaxis for up to 6 months. This breakthrough opens novel avenues of research into universal influenza vaccines.

## Figures and Tables

**Table 1:** Nbs reported in the treatment of viral infections.

Virus	Form	Target	Affinity (KD)	Function	Reference
Respiratory syncytial virus	Trimer	F protein	0.1 nM	Inhibiting viral fusion	[90]
	Dimer	F protein	1.8 nM		[91,92]
	Monomer	PreF protein	0.4–0.5 nM		[22]
	Monomer	PreF protein	<18.0 pM		[36]
	Monomer	PreF protein	84.0 pM		[37]
	Monomer	PreF protein	EC_50_: ~18 ng/mL		[93]
SARS-CoV-2	Monomer	RBD	38.6 nM	Inhibiting binding	[40]
	Monomer	RBD	<1.0 pM		[41]
	Monomer	RBD	3.3–8.2 nM		[43]
	Monomer	RBD	4.9 nM		[44]
	Monomer	RBD	4.0 nM		[45]
	Monomer	RBD	21.6 nM		[46]
	Monomer	RBD	1.9–22.2 nM		[94]
	Monomer	RBD	12.0 nM		[95]
	Monomer	RBD	57.0 nM		[48]
	Monomer	RBD	10.6 nM		[49]
	Monomer	RBD	1.0–35.5 nM		[50]
	Monomer	RBD	2.2 nM		[51]
	Monomer	RBD	64 nM		[96]
	Monomer	RBD	1.0 nM		[47]
	Monomer	RBD	1.4 nM		[58]
	Dimer	RBD	<0.1 nM		[52]
	Dimer	RBD	IC_50_: 1.5 nM		[53]
	Dimer	RBD	<1 nM		[24]
	Dimer	RBD	34.0 nM		[97]
	Trimer	RBD	0.5 nM		[25]
	Trimer	RBD	18 pM		[54]
	Trimer	RBD	<0.1 nM		[98]
	Trimer	RBD	41.0 nM		[56]
	Monomer Dimer Trimer Decameric	RBD	IC_50_: 2.4–6626 ng/mL IC_50_: 2.5–682.8 ng/mL IC_50_: 0.33–8202 ng/mL IC_50_: 0.18–18.7 ng/mL		[99]
	Monomer	RBD	0.5–3 nM		[100]
	Fc fused dimer	Conserved RBD residues	1.2–7.96 nM		[57]
	Monomer	N-terminal domain of S protein	1.4 nM	Inhibiting viral fusion	[58]
	Monomer Dimer Trimer	S2 domain	19–46 nM 5–10 nM 0.8–1.8 nM		[60]
Influenza virus	Monomer	H7 head region of HA	2.6 nM	Inhibiting binding	[64]
	Trimer	H5 head region of HA	IC_50_: 4.2 nM		[66]
	Monomer	IBV head region of HA	0.1–0.3 nM		[65]
	Monomer	H1 stem domain of HA	3.7–15.7 nM		[71]
	Monomer	H1 stem domain of HA	0.7 nM		[72]
	Monomer	H9 stem region of HA	6.9 nM		[101]
	Fc fused Dimer	H3 head region of HA	EC_50_: 0.02–0.92 nM		[67]
	Monomer	H1 head region of HA	EC_50_: 0.18–1.09 nM		[69]
	Monomer	Conserved lateral region of H7-HA head	IC_50_: 9 ng/mL		[70]
	Monomer	IAV and IBV stem region of HA	IC_50_: 10–100 nM		[102]
	Dimer	IAV and IBV stem region of HA	IC_50_: 1–100 nM		
	Dimer	H5 NA	0.4 nM	Inhibiting egress	[103]
	Monomer	M2	39.5 nM	Inhibiting uncoating	[104]
	Monomer	NP protein	N/A	Inhibiting replication	[105,106]
Poliovirus	Monomer	Capsid protein	6–84 nM	Inhibiting binding	[77,78,79]
Hepatitis B virus	Monomer	B surface antigen	EC_50_: 17.9–66.9 ng/mL	Inhibiting binding	[80]
Hepatitis C virus	Monomer	E2 glycoprotein	1–10 μg/mL	Inhibiting binding and transmission	[81]
	Monomer	NS3 Protease	N/A	Inhibiting replication	[107,108]
Human immunodeficiency virus	Monomer	gp120	IC_50_: <3 ng/mL	Inhibiting binding	[109]
	Monomer Multimer	gp120	0.2–4.4 nM IC_50_: 0.5–21.0 nM		[110]
	Dimer	gp41	0.15–29 nM		[111]
	Monomer Dimer	CXCR4	IC_50_: 13.6–82.0 nM IC_50_: 0.1–0.35 nM		[73]
	Monomer	CXCR4	6.2–7.7 nM		[74,75]
	Monomer	Rev protein	N/A	Inhibiting transcription	[112]
	Monomer	Nef protein	2 nM	Inhibiting critical biological activities of Nef.	[113]
Papillomavirus	Monomer	E7 oncoprotein	750 nM	Inhibiting binding	[82]
Rotavirus	Monomer	VP6 capsid protein	EC_50_: 1.6 ng/ml	Inhibiting binding	[83]
Ebola Virus	Monomer	Glycoprotein	55.3 nM	Inhibiting binding	[84]
Bunya virus	Multimer	Glycoprotein	ND_50_: <10 nM	Inhibiting binding	[114]
Chikungunya virus	Monomer	Glycoprotein E2	PRNT_50_: 2.4 ng/mL	Inhibiting binding	[85]
	Monomer	Glycoprotein E2	PRNT_50_: 45.6 nM		[86]
	Multimer	Glycoprotein E2	2.59–20.7 nM		[87]
Thrombocytopenia syndrome virus	Fc fused Nb	Glycoprotein N	IC_50_: 1.05 μg/mL	Inhibiting binding	[89]
Nipah virus	Monomer	Fusion protein	Binding energies: −3.73 kcal/mol	Inhibiting viral fusion	[88]

N/A, Not Available.

**Table 2:** Reported Nbs in the treatment of bacterial infections.

Bacteria	Form	Target	Affinity (KD)	Function	Reference
Enterotoxigenic *Escherichia coli*	Monomer	CfaE	IC_100_: 2.4–8 µM	Inhibiting colonization.	[127]
	Monomer	TssM	2–67 nM	Inhibiting toxin secretion	[138,139]
	Monomer	BtuCD-F	1–770 nM	Inhibiting nutrients uptake	[131]
*Acinetobacter baumannii*	Monomer	Bap	38 nM	Inhibiting colonization.	[128]
*Pseudomonas aeruginosa*	Monomer	Flagellin	2–5 nM	Inhibiting biofilm formation	[130]
*Campylobacter*	Monomer	Outer membrane protein	118 nM	Inhibiting colonization	[163]
Shiga toxin-producing *Escherichia coli*	Monomer	B subunit of toxin Stx2e	IC_50_: 8 nM	Neutralizing toxin	[141]
	Monomer Multimer	Stx2B	IC_50_: 0.5 nM IC_50_: 0.05 nM	Neutralizing toxin	[164]
	Multimer	Stx1B and Stx2B	Stx1 KD: 0.5 nM Stx2 KD: 0.004 nM	Neutralizing toxin	[142]
*Clostridium difficile*	Multimer	GTD, CPD and RBD of TcdA and TcdB	TcdA IC_50_: 100 pM TcdB IC_50_: 10 pM	Neutralizing toxin	[151]
	Multimer	RBD of TcdA and TcdB	TcdA KD: 2–290 nM TcdB KD: 2–24 nM	Neutralizing toxin	[152]
	Monomer	GTD CPD-DRBD DRBD CROPs	EC_50_: 110–350 nM EC_50_: <1 nM EC_50_: <1 nM EC_50_: 15.6–677.5 nM	Neutralizing toxin	[150]
	Monomer	CDTa ad CDTb	0.5–15 nM	Neutralizing toxin and inhibiting adherence	[156]
	Monomer	Surface layer proteins	3–6 nM	Inhibiting motility	[165]
*Bacillus anthracis*	Monomer	Domain 4 of PA	N/A	Neutralizing toxin	[158]
	Dimer	Domain 4 of PA and PA63 oligomer	IC_50_: 200 pM		[159]
	Dimer	LF and EF	10 pM		[160]
	Dimer	EA1	N/A	Inhibiting bacteria growth	[166]
*Helicobacter pylori*	Dimer	UreC	50 nM	Neutralizing toxin	[161]
	Dimer	UreC	20 nM		[162]

N/A, Not Available.

**Table 3:** Clinical trials of Nbs and monoclonal antibodies in the treatment of infectious diseases.

Category	Disease	Identifier	Biological	Clinical Trial Status	FDA Approval Dates
Nanobody	*Campylobacter* infection	NCT04182490	LMN-101	Phase II (completed)	
	Rotaviral diarrhoea	NCT01259765	VHH 203027	Phase II (completed)	
	Respiratory syncytial virus	NCT02979431	ALX-0171	Phase II (completed)	
Antibody	Respiratory syncytial virus	NCT05118386	RSM01	Phase I (completed)	
		NCT06551506	Nirsevimab	Phase IV (completed)	2023
		NCT00233064	Palivizumab	Phase IV (completed)	1998
		NCT04767373	Clesrovimab	Phase III (completed)	
	SARS-CoV-2	NCT05982704	Tixagevimab/Cilgavimab	Phase IV	2021
		NCT05780268	LY3819253	Phase III (completed)	2020
		NCT05502081	Casirivimab/Imdevimab	Phase IV (completed)	2020
	Influenza virus	NCT02603952	MEDI8852	Phase II (completed)	
		NCT04780321	JS016	Phase II (completed)	
	Hepatitis B virus	NCT05856890	HepB mAb19	Phase I	
	Human immunodeficiency virus	NCT02707861	Ibalizumab	Phase III (completed)	2018
		NCT02664415	VRC01	Phase II (completed)	
		NCT02588586	3BNC117	Phase II (completed)	
	Ebola virus	NCT06841614	Inmazeb	Phase III	2020
		NCT03719586	MAb114	Phase III (completed)	2020
		NCT03719586	ZMapp	Phase III (completed)	2017
	*Clostridium difficile* infection	NCT03880539	Bezlotoxumab	Phase IV (completed)	2016
	*Bacillus anthracis* infection	NCT02177721	Raxibacumab	Phase IV	2012
		NCT03088111	Obiltoxaximab	Phase IV	2016

## Data Availability

No new data were created or analyzed in this study.
